# The role of short chain fatty acids in appetite regulation and energy homeostasis

**DOI:** 10.1038/ijo.2015.84

**Published:** 2015-06-09

**Authors:** C S Byrne, E S Chambers, D J Morrison, G Frost

**Affiliations:** 1 Nutrition and Dietetic Research Group, Division of Diabetes, Endocrinology and Metabolism, Section of Investigative Medicine, Faculty of Medicine, Hammersmith Campus, Imperial College London, London, UK; 2 Stable Isotope Biochemistry Laboratory, Scottish Universities Environmental Research Centre, University of Glasgow, Glasgow, Scotland

## Abstract

Over the last 20 years there has been an increasing interest in the influence of the gastrointestinal tract on appetite regulation. Much of the focus has been on the neuronal and hormonal relationship between the gastrointestinal tract and the brain. There is now mounting evidence that the colonic microbiota and their metabolic activity have a significant role in energy homeostasis. The supply of substrate to the colonic microbiota has a major impact on the microbial population and the metabolites they produce, particularly short chain fatty acids (SCFAs). SCFAs are produced when non-digestible carbohydrates, namely dietary fibres and resistant starch, undergo fermentation by the colonic microbiota. Both the consumption of fermentable carbohydrates and the administration of SCFAs have been reported to result in a wide range of health benefits including improvements in body composition, glucose homeostasis, blood lipid profiles and reduced body weight and colon cancer risk. However, published studies tend to report the effects that fermentable carbohydrates and SCFAs have on specific tissues and metabolic processes, and fail to explain how these local effects translate into systemic effects and the mitigation of disease risk. Moreover, studies tend to investigate SCFAs collectively and neglect to report the effects associated with individual SCFAs. Here, we bring together the recent evidence and suggest an overarching model for the effects of SCFAs on one of their beneficial aspects: appetite regulation and energy homeostasis.

## Introduction

Obesity has become a global epidemic, with incidence rates of over 20% in the majority of western countries.^1^ It has been proposed that the current obesity epidemic may have been caused by a mismatch between the physiological mechanisms for maintaining energy balance, which evolved in response to ancestral diets, and the composition of the current western diet.^2^ Over the past several decades, the western diet has changed significantly with the popularity of ‘fast' and ‘convenience' foods rapidly increasing.^3^ Such foods are energy dense, have a low dietary fibre content and produce lower satiety and satiation signals than low-energy dense foods.^4^ This diet is markedly different to the historical low-energy dense, nutrient-poor diet that the human gut was adapted to over several millennia. Evidence suggests that for most of history the human lineage consumed more indigestible plant material, such as grasses, sedges and tubers, than is present in a typical western-style diet (>100 g per day dietary fibre compared with <15 g per day in the average modern-day diet), and is therefore likely to have contained a larger non-digestible component.^5, 6^

Some carbohydrates resist digestion in the upper gastrointestinal tract and reach the large bowel mainly intact where they are subject to fermentation by the resident bacteria. The human gut microbiota is composed of 10^13^ –10^14^ microorganisms, accounting for >1 kg of body weight.^7, 8^ The gut microbiota is continuing to emerge as a major determinant of obesity and its associated health complications.^9, 10^ However, as this topic is beyond the scope of the present review readers are referred to the excellent review by Holmes *et al.*^11^, which focuses on the composition and functional activities of the gut microbiota.

The principle products of the bacterial fermentation of non-digestible carbohydrates in the gut are short chain fatty acids (SCFAs), heat and gases.^12, 13, 14^ The process of bacterial fermentation serves as an energy harvest system for undigested material, rescuing energy that cannot be absorbed in the small bowel, and is used as a major energy source for some species. For example, lowland gorillas derive ~57% of their metabolisable energy from SCFAs, compared with 1.2–10% in humans from the average western diet.^15, 16, 17^ The main SCFAs produced by bacterial fermentation are acetate, propionate and butyrate, and are present in the approximate molar ratio of 60:20:20.^18^ It has been demonstrated that the consumption of soluble fermentable carbohydrates (FCs) increases the caecel content of SCFAs in animal models.^19, 20^ The rate, ratio and extent of SCFA production, however, is a complex interplay between FC type, microbiome diversity and activity, and gut transit time.^21, 22, 23, 24, 25^

Supplementing the high-fat diet of rodents with soluble FCs has been shown to protect against body weight and fat mass gain.^26, 27, 28^ Futhermore, research suggests that adult rodents who consume a weaning diet high in prebiotic fibre are protected against body weight gain when challenged with a western-style diet high in fat and sucrose.^29^ However, research carried out by Track *et al.*^30^ suggests that the beneficial effects of FC consumption are specific to adolescent rodents. In addition to improvements in body composition, a number of research studies in humans have reported associations between the consumption of FCs and improvements in glucose homeostasis, insulin sensitivity and blood lipid profiles, however, these beneficial effects were not present in young healthy adults.^31, 32, 33, 34, 35, 36^

Although it is known that greater FC consumption increases colonic SCFA production resulting in a wide range of health benefits, further research is needed to fully elucidate the molecular mechanisms by which SCFA mediate these effects. Published research often focuses on single mechanisms to explain the positive physiological effects associated with gut-derived SCFAs. However, we hypothesise that the beneficial effects reported are not the result of the activity of a single metabolic process on a specific tissue, but are more likely to be the result of the stimulation of a number of mechanisms activated in parallel. Here we review recent findings in this field and propose an interconnected picture of how SCFAs may affect appetite regulation and energy homeostasis.

## Materials and methods

**Figure 1:**
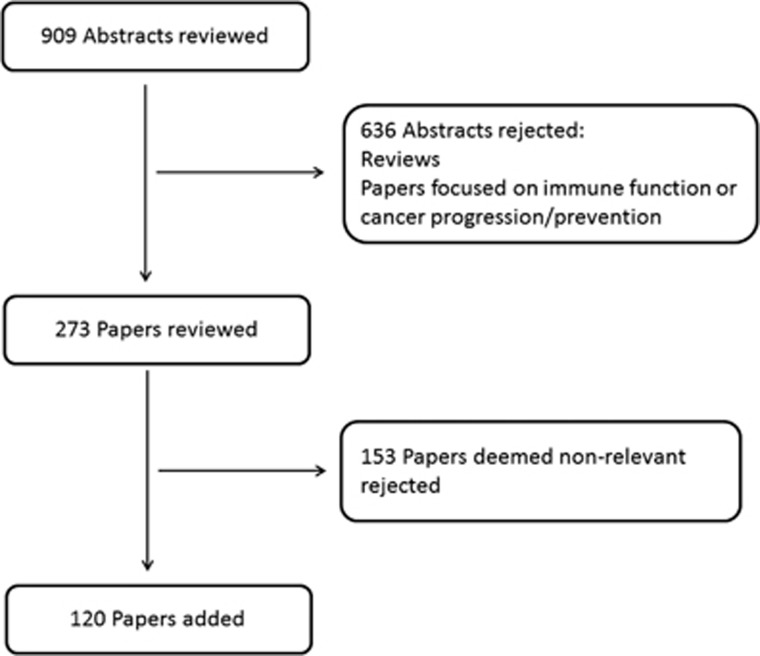
Flowchart outlining the methods used for research paper selection.

A review of the literature was conducted in 2014 using PubMed databases, with the following search terms: ‘short chain fatty acids' AND dietary fiber(MeSH terms), ‘short chain fatty acids' AND obesity(MeSH terms), ‘short chain fatty acids' AND appetite(MeSH terms), ‘short chain fatty acids' AND energy intake(MeSH terms), ‘short chain fatty acids' AND energy expenditure(MeSH terms), ‘short chain fatty acids' AND microbiota(MeSH terms), ‘short chain fatty acids' AND (‘free fatty acid receptor 3' OR ‘GPR41' OR ‘GPCR41') and ‘short chain fatty acids' AND (‘free fatty acid receptor 2' OR ‘GPR43' OR ‘GPCR43'). Reviews and research studies in which immune function or cancer progression/prevention were the primary focus were excluded as these topics were deemed beyond the scope of this review. Papers identified from the search were analysed by two of the authors and papers that were not relevant were rejected as shown in Figure 1. In total, 104 papers were identified as containing relevant primary evidence.

### SCFAs and free fatty acid receptor signalling

In 2003, three independent research groups discovered that the orphan G-protein-coupled receptors, GPR43 and GPR41, were activated by SCFAs.^37, 38, 39^ These receptors have since been renamed free fatty acid receptor 2 and 3 (FFA2 and FFA3; formerly GPR43 and GPR41, respectively). Acetate and propionate are the most potent activators of FFA2, whereas FFA3 is activated in the affinity order propionate>butyrate⩾acetate.^37, 38, 39^ However, results of studies using animal models must be noted with caution owing to interspecies variability. Hudson *et al.*^40^ reported that in mice FFA2 and FFA3 have equal affinity for acetate and butyrate, whereas FFA3 has higher affinity for propionate than FFA2.

FFA2 and FFA3 are both widely expressed throughout the small intestine and colon.^37, 41, 42^ FFA2 and FFA3 mRNA have also been discovered in areas other than the gut, which lead to the assumption that SCFAs are likely to have beneficial effects on tissues and organs beyond the gut. FFA2 mRNA has been detected in immune cells, skeletal muscle, heart, spleen and adipose tissue,^37, 42, 43, 44^ whereas the expression of FFA3 appears to be more widespread and has been detected in adipose tissue, peripheral blood mononuclear cells, pancreas, spleen, bone marrow and lymph nodes.^37, 38, 42^ However, reports investigating the expression of both SCFA receptors in adipose tissue have proven to be inconsistent.^37, 43, 45, 46, 47^

### SCFAs and energy intake in animal models

The addition of FCs to the diets of animals has been shown to reduce energy intake.^26, 27, 48, 49^ Several studies have investigated the effect of FCs on feeding motivation, however, the results have been equivocal.^50, 51^ Results from studies published by our research group have shown that FCs increase manganese-enhanced magnetic resonance imaging signals in the appetite centres of the hypothalamus, further suggesting a satiating effect.^52, 53^ In addition, feeding FCs, as well as SCFAs themselves, have been associated with an increase in the circulating concentrations of the anorectic gut hormones, glucagon-like peptide-1 (GLP-1) and peptide YY (PYY).^26, 27, 29, 48, 54, 55, 56, 57, 58, 59^ GLP-1 and PYY are produced by L cells, which are present throughout the gastrointestinal tract, with the highest concentrations observed in the distal ileum and colon, and are released in response to food intake.^60, 61^ Peripheral infusions of these gut hormones have been shown to cause a reduction in energy intake and have thus become the target of many anti-obesity therapies.^62, 63, 64, 65^

The discovery of the co-expression of FFA2 and FFA3 in GLP-1 and PYY releasing enteroendocrine L cells has prompted suggestions that the detection of SCFAs in the colon may be responsible for triggering the release of these gut hormones.^54, 66, 67^ This theory is supported by reports that FFA3 knock-out (KO) mice demonstrate an impaired PYY expression^68^ and that FFA2 KO mice exhibit reduced GLP-1 concentrations *in vivo* and in response to SCFA *in vitro*.^54^ Furthermore, FC supplementation has been shown to increase the densities of FFA2-positive enteroendocrine cells in parallel with GLP-1-containing cells.^69^ However, it is currently unclear whether this is because of a SCFA-stimulated increase in cell proliferation or an increase in the expression of SCFA receptors in the gut epithelium.

It has recently been reported that propionate stimulates the secretion of both GLP-1 and PYY from wild type (WT) primary murine colonic crypt cultures, an effect that was significantly reduced in FFA2 KO mice cultures.^70^ In addition, intra-colonic infusions of propionate reportedly increased both GLP-1 and PYY levels in jugular vein plasma *in vivo*, an effect that was not present in FFA2 KO mice. These data further support the mounting evidence that FCs stimulate the secretion of GLP-1 and PYY. Additional evidence suggests a role for propionate in the modulated expression of FFA2, PPAR-γ (peroxisome proliferator-activated receptor gamma), Fiaf and histone deacetylases.^71^

Our research group recently investigated the role of the most abundant SCFA, acetate, in central appetite regulation in mice.^72^ In line with recent observations, we noted that dietary supplementation with the FC inulin causes a significant reduction in energy intake and weight gain.^26, 73^ In addition, we investigated the effect of intravenous and colonic infusions of ^11^C-acetate *in vivo* using positron-emission tomography-computed tomography scanning and found that although the majority of ^11^C-acetate tracer was absorbed by the heart and liver, a small amount crossed the blood-brain barrier (~3%) and was taken up by the brain. We subsequently confirmed that acetate induces hypothalamic neuronal activation in the arcuate nucleus following intraperitoneal administration, suggesting that acetate itself is an anorectic signal.

That FCs reportedly have beneficial effects on energy homeostasis but also increase energy harvest appears counterintuitive. It has been suggested that the metabolisable energy gained from SCFAs via the colonic fermentation process of non-digestible carbohydrates may outweigh the beneficial effects associated with their consumption. Indeed, Isken *et al.*^74^ demonstrated that long-term consumption (45 weeks) of soluble guar fibre significantly increased both body weight and markers of insulin resistance in mice, when compared with controls, despite a comparable dietary energy intake in both groups. Their data provides evidence that increased SCFA production in rodents, which significantly contributes to digested energy, may outweigh the short-term beneficial effects of soluble fibre consumption. These results may be explained by research carried out by Track *et al.*^30^, which reported that feeding adolescent rats guar gum results in a reduction in food intake and weight gain and improved glucose tolerance. However, these beneficial effects were only observed in adolescent rats, when compared with controls, and were absent in adults.

### FCs and energy intake in humans

As discussed earlier, evidence suggests that hominin's diets consisted of mainly vegetable matter and would have had a large fermentable component.^5^ It is highly likely that this large FC consumption would therefore have stimulated gut hormone release, slowing gastric emptying and small intestinal transit. It is possible that this was advantageous as it would have increased the energy harvest from nutritionally poor food during periods when it was a major struggle for hominins to meet their energy demands. However, it is likely that this physiological adaptation is underutilised by humans in the developed world owing to wider food availability and the lower FC content of the average westerner's diet.^15^ Behall *et al.*^75^ suggest that the fermentation process significantly contributes to digestible energy when amounts of >20 g non-digestible carbohydrates are consumed. It has been demonstrated that overweight and obese individuals have higher faecal SCFA concentrations than their lean counterparts.^24, 76^ The results suggest that these individuals produce more colonic SCFA, indicating an increased microbial energy harvest in obesity.^76^ However, *in vitro* fermentations using faecal samples from obese and lean individuals displayed no difference in total SCFA production.^77^

Although there is clear evidence from a number of small animal studies that the addition of FCs to the feed of high-fat-fed animals results in improvements in body weight and composition,^26, 27, 28^ translation to humans has proven inconsistent. This may be owing to the relatively small amount of FC used in human experimental diets compared with animal studies (1.5% and >5% of total energy intake, respectively). Large amounts of FCs are generally not well tolerated as they are associated with undesirable gastrointestinal effects, resulting in the use of lower doses in human research trials.

However, a number of notable acute and long-term supplementation studies have been successfully carried out in humans. Archer *et al.*^78^ reported that replacing fat with an acute dose of inulin (24 g) at breakfast results in lower energy and fat intake throughout the day, although gut hormone concentrations were not reported. Nilsson *et al.*^79^ demonstrated that consuming an evening meal consisting of FCs significantly increases circulating PYY concentrations and decreases ghrelin concentrations at breakfast. Our research group recently carried out a dose-finding study and demonstrated that increased circulating PYY concentrations and appetite suppression occurs only with an acute dose of >35 g per day of inulin, suggesting the need for large doses in order to induce appetite suppression.^80^ Evidence from long-term studies suggest that supplementation (2–12 weeks) with oligofructose (16–30 g per day) significantly increases feelings of satiety and reduces feelings of hunger, reduces energy intake, increases the total area under the curve for PYY and reduces total area under the curve for ghrelin, an orexigenic hormone.^81, 82, 83^ A 1-year study investigating supplementation with high-wheat fibre also resulted in both an increase in SCFA production and GLP-1 secretion.^84^ The authors note that these changes took 9–12 months to develop, suggesting that it may take up to a year for the gut microbiota to adapt to the extra fermentable content of the diet. However, data from a recent study showed that short-term dietary change alters both the microbial community structure and gene expression of the human gut microbiome, rapidly and reproducibly.^85^ Thus, the optimum time period for adaptation to a high FC diet is, at the present time, unclear.

Our research group recently investigated the role of propionate in appetite regulation. We demonstrated that propionate significantly stimulates the release of PYY and GLP-1 from human colonic cells.^86^ Next, we produced a novel system, inulin-propionate ester, whereby propionate is conjugated by an ester linkage to inulin, a carrier molecule. The ester linkage is broken down by bacterial fermentation, which results in the delivery of propionate directly to the colon. When administered acutely, we found that inulin-propionate ester significantly increased postprandial PYY and GLP-1, and reduced energy intake by ~14% at a buffet meal. Furthermore, after a 24-week supplementation period we demonstrated that inulin-propionate ester significantly reduced weight gain in overweight adults.

### Beneficial effects associated with SCFA production independent of food intake

The importance of SCFAs to energy metabolism has been further emphasised in recent studies where germ-free mice have received gut microbiota transplants. These investigations highlight that the transfer of gut microbiota compositions, which produce different levels of SCFAs in the colon, influence body weight gain and adiposity.^87, 88^ For example, it has been shown that the transplantation of the faecal microbiota of twins discordant for obesity to germ-free mice results in a similar phenotype in the recipient mice.^87^ It was noted that the lean mice demonstrated significantly increased caecal propionate and butyrate contents when compared with their obese counterparts. Their data suggest that the increased weight gain observed in the obese mice was not caused by an increased energy harvest by the gut microbiota and suggests that instead, SCFAs inhibit the fat accumulation associated with obesity. Similarly, the faecal transplantation of mice that have undergone Roux-en-Y gastric bypass (RYGB) surgery to germ-free mice has been shown to result in weight loss and reduced fat mass in the RYGB-recipient mice.^88^ In addition, the RYGB-recipient mice exhibited a relatively greater production of propionate and lower production of acetate when compared with mice that received the faecal microbiota of those that had undergone sham surgery. The beneficial change in body composition observed in RYGB-recipient mice may be owing to the beneficial effects associated with the SCFA production profile of these mice. The authors suggest that the reduced levels of acetate would result in decreased lipogenesis and that the increased levels of propionate would assist in the inhibition of acetate conversion into lipid in the liver and adipose tissue.^43, 89, 90^ The metabolic effects noted in these studies were not associated with any significant change in energy intake,^87, 88^ suggesting that the positive effects on energy balance observed may be a result of a change in energy utilisation and expenditure.

A recent study carried out by Remely *et al.*^91^ demonstrated a lower methylation status in the promoter region of the FFA3 gene in the blood of both obese and type 2 diabetics, when compared with lean individuals. The researchers hypothesise that this is owing to compositional differences in the gut microbiota and therefore different SCFA profiles.

### SCFAs, energy expenditure and substrate metabolism

Although the consumption of FCs and SCFAs have been associated with a reduction in energy intake, there is also evidence that SCFAs may increase energy expenditure. SCFAs have been shown to increase the rates of oxygen consumption, enhance both adaptive thermogenesis and fat oxidation and increase mitochondrial function in rodents.^73, 92^ Marsan and McBurney^93^ also demonstrated that the oxidation of all three principle SCFAs was significantly higher for colonocytes isolated from rodents who had consumed a high fibre diet for 14 days. Gao *et al.*^73^ also investigated the expression of two thermogenesis-related genes, *PGC-1α* and *UCP-1*, and discovered that the mRNA and expression of both genes were upregulated in those whose diets were supplemented with butyrate. Furthermore, consuming a diet high in whole-grain foods has been shown to decrease urinary excretion of markers of protein catabolism which was associated with an increase in SCFA production.^94^ In addition, it has been demonstrated that SCFAs can be used as an energy source for protein gain when pigs are fed below their energy requirements.^95^

Evidence from several research studies indicates that the SCFA receptors FFA2 and FFA3 may have a critical role in energy homeostasis. It has been demonstrated that FFA3 KO mice exhibit a reduced energy expenditure, compared with WT mice, despite having matching physical activity levels.^92, 96^ In addition, Kimura *et al.*^92^ reported that treatment with propionate increases the rate of oxygen consumption in WT mice, a result that was not present in FFA3 KO mice. SCFAs were subsequently shown to stimulate sympathetic nervous system activity directly through FFA3 at the sympathetic ganglion, thereby controlling energy expenditure.^92^ In addition, recent evidence suggests that propionate binds FFA3 in the periportal afferent system to induce intestinal gluconeogenesis (IGN) via a gut–brain neural circuit.^97^ Similarly, it has been reported that FFA2 KO mice exhibit a reduction in energy expenditure when fed a high-fat diet, compared with WT mice, and are obese despite a similar physical activity level.^98^ In contrast, mice with adipose-specific overexpression of FFA2 exhibited an increase in energy expenditure. Interestingly, the FFA2 KO mice had a higher RER than WT mice, which suggests a reduced capacity to oxidise fat, whereas mice with adipose-specific overexpression of FFA2 had a lower RER than WT mice. The authors note that their results indicate that FFA2 activation increases energy expenditure and the capacity to oxidise fats via the suppression of fat accumulation and adipose tissue insulin signalling.

Research suggests that SCFAs and their receptors, FFA2 and FFA3, may have a critical role in maintaining energy homeostasis. However, there is currently no known study that has specifically investigated the effect of FC consumption on energy expenditure in humans and would make for an interesting line of investigation.

### SCFAs and hepatic metabolism

There is evidence that feeding rodents a diet supplemented with FCs or SCFAs results in reduced intrahepatocellular lipid levels, liver triglyceride and cholesterol content, hepatic cholesterol synthesis and hepatic glucose production.^52, 53, 97, 99, 100, 101^ SCFAs are absorbed from the intestinal lumen into the portal vein and subsequently enter the hepatic blood flow. As butyrate is the preferred fuel for colonocytes, the majority of butyrate produced in the gut is rapidly utilised at the epithelium.^18, 102^ In contrast, the majority of propionate and acetate produced in the gut is absorbed and drains into the portal vein.^103^ Cummings *et al.*^18^ investigated SCFA distribution in sudden death victims and demonstrated that the majority of butyrate and propionate present in the portal vein is extracted by the liver and subsequently metabolised (86 and 94%, respectively), with a small amount of remaining propionate and butyrate entering venous blood. Bloemen *et al.*^102^ reported that liver uptake of acetate was not significant. These results may suggest that any hepatic changes associated with FC consumption or SCFA administration are largely because of the metabolism of propionate by the liver. It is known that propionate is a gluconeogenic substrate and inhibits the utilisation of acetate for lipid and cholesterol synthesis.^89, 104^ Therefore, potential upregulation of this pathway after FC consumption is likely to be responsible for any observed changes in hepatic structure or function.

A number of studies have reported that FC consumption in humans beneficially affects serum cholesterol and triglyceride concentrations, and reduces hepatic lipogenesis.^31, 35, 105, 106, 107^ In addition, our research group recently demonstrated a significant reduction in the intrahepatocellular lipid levels of overweight adults meeting the criteria for non-alcoholic fatty liver disease after a 24-week increase in colonic propionate concentrations.^86^ Furthermore, SCFAs may have an indirect benefit on hepatic metabolism through their effect on gut hormone secretion. In particular, GLP-1 has been shown to modulate physiological mechanisms responsible for free fatty acid accumulation in the liver and reduce hepatic steatosis.^108^

### SCFAs, glucose uptake and gluconeogenesis

The consumption of FCs has been associated with an improvement in glucose homeostasis, although the evidence in humans is inconsistent. Propionate is gluconeogenic and has been shown to produce a dose-dependent increase in blood glucose concentrations in humans.^89, 104, 109^ Again, the notion that FC consumption may have beneficial effects on glucose homeostasis appears paradoxical. However, it has been demonstrated that SCFAs have no significant effect on glucose metabolism in healthy men.^110^ In addition, it has been shown that propionate-supplementation induces a reduction in fasting blood glucose in rats.^111^

The intestine has recently been established as a gluconeogenic organ and it has been reported that IGN promotes metabolic benefits and regulates energy and glucose homeostasis.^112, 113^ Delaere *et al.*^114^ later demonstrated that a portal vein glucose sensor is activated by IGN and transmits signals to the brain via the peripheral nervous system, which initiates these beneficial effects. It has been reported that both propionate and butyrate stimulate IGN.^97^ Although butyrate was found to directly activate the expression of IGN genes in enterocytes, propionate itself was shown to act as a substrate for IGN. In addition, rats fed a SCFA- or a FC-supplemented diet displayed a significantly lower weight gain, reduced adiposity, improved glucose control and reduced hepatic glucose production when compared with the control group. It was noted that this improvement in glucose tolerance involved both gut–brain communication and IGN, and that none of the reported metabolic benefits were present in mice lacking the catalytic subunit of a key enzyme involved in IGN, intestinal glucose-6-phosphatase, despite a similar shift in the gut microbiota composition. These data suggest that IGN has a major role in mediating the beneficial effects associated with the consumption of FCs.

### SCFAs and adipocytes

It has been observed that FC consumption protects against fat mass development.^26, 27, 28^ All three principle SCFAs have also been shown to protect against diet-induced obesity.^55^

A number of studies have reported that treatment with the SCFAs, propionate and acetate increases the expression of leptin, a potent anorectic hormone, in adipocytes *in vitro*, whereas butyrate has been shown to have no effect.^43, 45, 46^ In addition, propionate has been shown to increase plasma leptin concentrations in mice *in vivo*^45^ and stimulate mRNA expression in human adipose tissue.^115^ Xiong *et al.*^45^ reported that this SCFA-stimulated increase in leptin expression in adipocytes is mediated by FFA3. However, not all reports regarding the body composition of FFA3 KO mice have been consistent.^55, 68, 96^ Furthermore, a number of contradictory reports suggesting that the expression of FFA3 cannot be detected in adipose tissue have been published, indicating that the SCFA-stimulated increase in leptin expression is not mediated by FFA3.^43, 46^ Zaibi *et al.*^46^ suggest that the SCFA-stimulated increase in leptin expression is mediated by FFA2 and that the downregulation of FFA2 in FFA3 KO mice is responsible for the reduction in SCFA-stimulated leptin secretion observed in FFA3 KO mice. However, Frost *et al.*^47^ failed to demonstrate a significant effect of SCFAs on leptin secretion in adipocytes.

Kimura *et al.*^98^ recently demonstrated that SCFA-mediated activation of FFA2 suppresses insulin signalling within adipocytes, which results in the inhibition of fat accumulation within adipose tissue and the promotion of metabolism of unincorporated glucose and lipids in other tissues. In addition, it was reported that FFA2 KO mice were obese on a normal diet, which was further enhanced by a high-fat diet, that adipose-specific FFA2 transgenic mice had a significantly lower body weight than WT mice and that mice overexpressing FFA2 in their adipose tissue remained lean even when consuming a high-fat diet. The researchers suggest that FFA2 may act as a sensor for excessive dietary energy, controlling energy utilisation and maintaining metabolic homeostasis. However, these observations are not supported by Bjursell *et al.*^116^ who reported that FFA2-deficient mice consuming a high-fat diet exhibit a reduction in body-fat mass and increase in lean body mass. In addition, it has been shown that all three principle SCFAs enhance the degree of adipocyte differentiation^43, 117^ and that propionate and acetate inhibit lipolysis.^43^ Hong *et al.*^43^ also demonstrated that propionate increases the expression of FFA2 during adipocyte differentiation and causes an upregulation of PPAR-γ2. The authors suggest that these results indicate the involvement of FFA2 in the lipid accumulation pathway. This is further supported by Ge *et al.*^118^ who reported that acetate and propionate inhibit adipose tissue lipolysis in a mouse model via FFA2 resulting in a reduction in plasma free fatty acid concentrations. Hosseini *et al.*^119^ demonstrated that propionate increased the gene expression of adiponectic receptors 1 and 2 in the adiponectin system.

It has also been suggested that FFA3 may have a role in insulin-stimulated glucose uptake. Han *et al.*^120^ reported that propionate and valerate enhance insulin-stimulated glucose uptake in adipocytes which appeared to be mediated via FFA3.

Although data from animal studies suggest that SCFAs and the activity of their receptors, FFA2 and FFA3, may have an inhibitory effect against weight gain, there is currently a lack of evidence to support this hypothesis in humans. As acetate and propionate are the most potent activators of FFA2 it seems likely that these SCFAs are responsible for any adipocyte-related changes observed after FC consumption or SCFA administration. However, as acetate circulates at a higher concentration than both butyrate and propionate, it seems the most likely SCFA to directly influence adipose tissue.^18^

## Conclusion

A significant body of evidence suggests that SCFAs have a beneficial role in appetite and energy homeostasis. However, as the majority of research comes from animal models, caution when translating this evidence to humans is necessary. Thus, there is an urgent need for human data to support the mechanistic data being reported. One major issue is that large amounts of FCs are generally not tolerated well by humans, which results in a relatively small amount of FCs being used in human experimental diets when compared with animal studies. Therefore, effective strategies that replicate the changes in SCFA profiles seen in animal studies, either via dietary or pharmacological means, may have the potential to translate the beneficial effects observed in animal studies to man.

**Figure 2:**
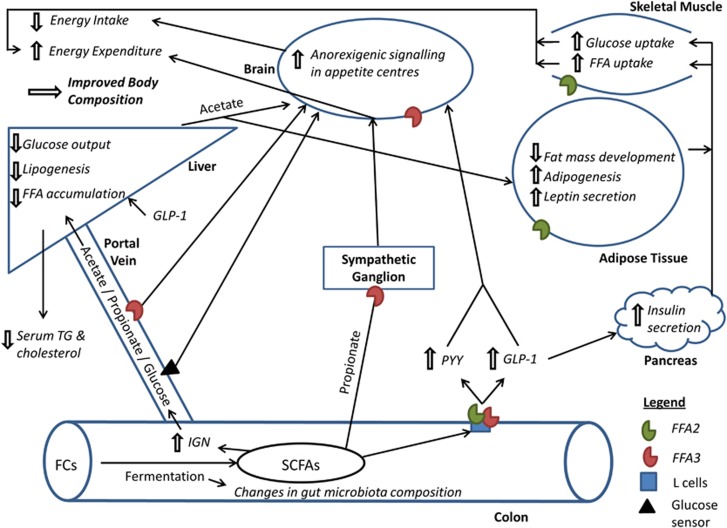
An overarching model for the beneficial effects of colonic SCFA production on appetite regulation and energy homeostasis. FCs, fermentable carbohydrates; FFA, free fatty acids; FFA2, free fatty acid receptor 2; FFA3, free fatty acid receptor 3; GLP-1, glucagon like peptide-1; IGN, intestinal gluconeogenesis; PYY, peptide YY; SCFAs, short chain fatty acids; TG, triglyceride.

In conclusion, it is evident that the administration of FCs and their breakdown products, SCFAs, have positive effects on host physiology. However, the majority of recent publications have investigated the effect of SCFAs on one particular tissue or metabolic process and have failed to look at the body system as a whole. Here, we propose that SCFAs have a number of metabolic processes, which are activated in parallel, that affect energy homeostasis and appetite regulation (summarised in Figure 2). Furthermore, the site-specific uptake of SCFA across the gut–liver–peripheral tissue axis suggests selectivity in the effect of individual SCFA. It is only by bringing these effects together that the true impact of SCFAs on host energy homeostasis can be seen.
